# Sequential Recovery of Non-Polar and Polar Compounds from Spent Coffee Grounds Using scCO_2_ Followed by SWE and UAE

**DOI:** 10.3390/foods15193469

**Published:** 2026-09-28

**Authors:** Aleksandra Gavarić, Senka Vidović, Anastasia Loukri, Eleni Zymvrakaki, Ioannis Mourtzinos, Siniša Simić, Mirjana Sulejmanović

**Affiliations:** 1 Faculty of Technology Novi Sad, University of Novi Sad, Boulevard cara Lazara 1, 21000 Novi Sad, Serbia; cvejina@uns.ac.rs (A.G.); senka.vidovic@uns.ac.rs (S.V.); sinisa.simic@uns.ac.rs (S.S.); 2 Department of Food Science and Technology, School of Agriculture, Aristotle University of Thessaloniki, 54124 Thessaloniki, Greece; loukriap@agro.auth.gr (A.L.); elenzym@agro.auth.gr (E.Z.); mourtzinos@agro.auth.gr (I.M.)

**Keywords:** spent coffee grounds, valorization, caffeine, lipids, HPLC, GC/FID

## Abstract

Spent coffee grounds (SCG), a solid by-product generated during coffee preparation, are produced in substantial quantities yet remain largely underexploited. The valorization of SCG offers significant environmental, economic, and social advantages, contributing to the advancement of a circular bioeconomy. In this study, SCG valorization consisted of two steps. In the first step, the SCG was depleted by supercritical CO_2_ at 250 bar for 4 h, and by using two different temperatures: 40 °C and 60 °C. In the second step, the defatted SCG was subjected to subcritical water extraction at different temperatures, and ultrasound-assisted extraction at both different amplitudes and temperatures. The objective of this study was to evaluate a sequential extraction approach for the recovery of fatty acid methyl esters (FAMEs) and the subsequent maximization of caffeine extraction from SCG. The FAME profile was determined in ten supercritical fluid extracts and two Soxhlet extracts, while caffeine content was evaluated in five subcritical water extracts and six ultrasound-assisted extracts obtained from SCG, previously exhausted by supercritical CO_2_ extraction. The highest caffeine content was present in a subcritical extract obtained at 180 °C, while the most abundant fatty acids found in all the tested extracts were linoleic acid (43.79–54.15%) and palmitic acid (32.73–45.85%). The predominance of linoleic and palmitic acids in the obtained extracts suggests their potential application as feedstocks for biodiesel production and oleochemical industries. Furthermore, the high content of linoleic acid, an essential polyunsaturated fatty acid, may enhance the value of these extracts for cosmetic and nutraceutical applications.

## 1. Introduction

Global food supply chain waste generation is one of the most significant environmental, social, and techno-economic challenges of the modern world. According to the latest report by the United Nations Environment Programme (UNEP)—Food Waste Index Report 2024, approximately 1.05 billion tonnes of food were wasted globally in 2022, which corresponds to one-fifth (19%) of all food available to consumers at the retail, food service, and household levels [1]. In addition to the aforementioned consumption waste, the Food and Agriculture Organization of the United Nations estimates that an additional 13.2% of food is lost in the supply chain from post-harvest up to (but not including) retail, bringing total losses in the food system close to one-third of global production [2]. Households are responsible for 60% of the stated amount (631 million tonnes), while the food service sector generates around 28%—or 290 million tonnes of food waste annually, averaging 36 kg per capita [1].

According to EUROSTAT statistics [3], the food service sector (NACE Rev. 2, Section 1) accounted for approximately 7 million tonnes of food waste in the EU in 2023, representing 11% of the total amount generated, whereas the retail sector contributed nearly 5 million tonnes, corresponding to 8% of the overall food waste stream. As part of the new revision of the Waste Framework Directive, the EU has set legally binding targets to reduce food waste by 30% per capita in retail, food service, and households by 2030 [4,5]. Furthermore, the European Commission identifies cafés, fast-food restaurants, and hotels as sub-sectors with the highest potential for waste reduction through prevention, the valorization of inedible parts, and digital tracking systems. The significance and volume of food waste generated in hospitality establishments is underscored by the fact that approximately 3.33 million tonnes of coffee were served across EU countries in 2022. It is estimated that one tonne of roasted coffee beans produces 650 kg of spent coffee grounds (SCG) [6,7]. Consequently, the European coffee consumption sector generates over 2 million tonnes of wet SCG annually. On a global scale, the annual production of SCG is estimated at 6–8 million tonnes [8].

The global coffee market is dominated by two major species, Arabica and Robusta. Arabica accounts for approximately 60% of worldwide coffee production and is generally regarded as a premium, higher-value coffee, predominantly used in the roasted and ground coffee sector. It is also commonly blended with Robusta to improve the quality of instant coffee products. Robusta, typically characterized by lower market value and quality, is primarily utilized in instant coffee manufacturing and coffee blends. Nevertheless, its incorporation into roast coffee blends, traditionally dominated by Arabica, has increased steadily in recent years (https://www.fao.org/markets-and-trade/commodities-overview/beverages/coffee/en, accessed on 15 July 2026).

SCG’s high content of lipids, polysaccharides, proteins, and phenolic compounds makes SCG an attractive feedstock for biorefinery and valorization approaches [9,10,11,12]. SCG is known to be particularly abundant in lipids and fatty acids. Therefore, supercritical CO_2_ extraction (scCO_2_) was applied to recover these compounds. Residual SCG biomass was further utilized for obtaining more polar bioactive compounds, particularly caffeine, also using green extraction techniques, subcritical water extraction (SWE) and ultrasound-assisted extraction (UAE) with 50% ethanol as the extraction solvent. Besides solid–liquid extraction, SCG has already been exposed to green solvents in the past. The most widely used green extraction technologies for SCG, particularly for the recovery of phenolic compounds, chlorogenic acids, flavonoids, and caffeine, are UAE and, less frequently, microwave-assisted extraction (MAE) [13,14,15,16,17]. In this way, valuable compounds were isolated, and the material left after extraction of polyphenolics could be safely discarded, while still not causing negative environmental impact, as indicated by the release of chemicals such as caffeine, polyphenols, and tannins into the environment [18,19,20,21].

UAE is the most widely applied green extraction technology for SCG due to its simplicity, cost-effectiveness, and high extraction efficiency. This technique enhances mass transfer through acoustic cavitation, facilitating solvent penetration into the biomass matrix and promoting the release of intracellular compounds. As a result, UAE enables shorter extraction times and lower solvent consumption compared to conventional extraction methods. Furthermore, it operates under relatively mild conditions, reducing the risk of thermal degradation of sensitive bioactives such as phenolics and caffeine. Its compatibility with environmentally friendly solvents, including water and aqueous ethanol, together with its ease of implementation and scalability, makes UAE an attractive approach for the sustainable valorization of SCG [13,22,23].

SWE has become increasingly popular in biomass valorization and biorefinery concepts over the last decade. This is mainly due to its environmentally friendly nature, as it uses water instead of organic solvents, while still providing high extraction efficiency for a broad range of bioactive compounds. In addition, the physicochemical properties of water can be tuned by adjusting temperature and pressure, enabling the selective recovery of phenolics, caffeine, carbohydrates, and other valuable compounds from coffee matrices. SWE is also considered economically attractive, scalable, safe, and compatible with food, nutraceutical, and pharmaceutical applications [24,25,26]. Recently, Karprakhon et al. [27] introduced high-pressure carbon dioxide into SWE to create a unique extraction environment that enhances efficiency through multiple mechanisms. The dissolution of CO_2_ in subcritical water led to the formation of carbonic acid, creating a mildly acidic extraction medium that enhanced cell wall disruption and increased the solubility of phenolic compounds. As a result, significantly higher total phenolic contents and antioxidant activities were achieved compared to conventional SWE, demonstrating the potential of CO_2_-assisted SWE for the efficient recovery of bioactive compounds from SCG.

The main objective of this study was to improve the extraction conditions for obtaining a comprehensive fatty acid methyl esters (FAMEs) profile and sequentially maximizing caffeine recovery by integrating green extraction techniques, scCO_2_ in the first stage for non-polar compound isolation, and as a pretreatment aimed at modifying the SCG matrix and facilitating the subsequent recovery of caffeine by aqueous extraction techniques, and UAE and SWE in the second stage.

## 2. Materials and Methods

### 2.1. Materials

Spent coffee grounds (SCG), generated during the preparation of commercially available Arabica coffees, were collected from several local coffee shops and restaurants, dried until achieving a moisture content of 3% *w*/*w*, and used as the raw material for subsequent extraction experiments. After drying, the SCG samples were stored in sealed plastic bags in a desiccator at room temperature until further analysis. Before extraction, the samples were homogenized to ensure sample uniformity. The mean particle size was 0.18–0.25 mm.

### 2.2. Supercritical CO_2_ Extraction (scCO_2_)

The extractions were performed using a laboratory-scale high-pressure extraction plant (extractor vessel with a heating jacket for the heating medium (with internal volume 200 mL, maximum operating pressure of 700 bar), separator with a cooling jacket for the cooling medium (with internal volume 200 mL and maximum operating pressure of 250 bar)) (NOVA-Swiss, Effretikon, Switzerland). For each extraction, 50 g of SCG were used (particle size of 0.18–0.25 mm). The pressure was kept constant at 250 bar. The flow of CO_2_ was measured using a mechanical flow meter (Q-Flow 140 by Vötglin Instruments GmbH, Muttenz, Switzerland). The impact of two different temperatures of 40 °C (sample marked as C1) and 60 °C (sample marked as C2) was examined. For a better understanding of the dynamics of the extraction procedure, fractionation was performed in accordance with our previous studies [28]. Fractionation of the scCO_2_ extraction included 1 h, 2 h, 3 h, and 4 h extraction yields (EY), with two temperatures applied: 40 °C and 60 °C. The samples were collected separately. The collected fraction labels were as follows: the extracts obtained at the lower temperature (40 °C) from 1 h to 4 h were marked C3–C6. The samples marked C6–C10 represent fractions achieved after 1 h, 2 h, 3 h, and 4 h of extraction at the higher temperature (60 °C).

$$EY\;(\%)=\frac{\mathrm{mass}\;\mathrm{of}\;\mathrm{the}\;\mathrm{obtained}\;\mathrm{extract}\;(g)}{\mathrm{mass}\;\mathrm{of}\;\mathrm{the}\;\mathrm{SCG}\;(g)}*\;100$$

### 2.3. Soxhlet Extraction

Soxhlet extraction served as a reference technique for the isolation of non-polar compounds. Soxhlet extraction was used for the determination of the total lipid content of SCG, and it was performed for 6 h using the solvent. The solid-to-solvent ratio (S/L) was 1:10 g/mL. Following the extraction, the solvent was removed under vacuum. The experiments were carried out in duplicate, and the results of extraction yields (EY) were expressed as g total lipids/100 g of SCG. Soxhlet extracts were designated as C11, and the EY was 12.6%, with two replicates (*n* = 2).

### 2.4. Subcritical Water Extraction (SWE)

SWE was performed using a high-pressure extractor (Parr Instrument Company, Moline, IL, USA). SWE was performed only with the material pretreated by scCO_2_, and the extraction procedure included temperatures of 120 °C, 140 °C, 160 °C, 180 °C, and 200 °C. Briefly, 7 g of the SCG that was previously extracted with scCO_2_ was treated with 140 mL of subcritical water (S/L ratio 1: 20 g/mL) at 2 MPa pressure, using stirring at 1000 rpm. The extraction time was kept constant at 15 min; the time needed for heating was approximately 5 min, and for cooling was 10 min. The obtained extracts were filtered under vacuum and stored at 4 °C until analysis. Total EY was expressed as a percentage as mg/mL. Samples obtained by subcritical water extraction of SCG, previously exhausted by scCO_2_, were designated as C12 (120 °C), C13 (140 °C), C14 (160 °C), C15 (180 °C) and C16 (200 °C).

### 2.5. Ultrasound-Assisted Extraction (UAE)

Ultrasound-assisted extraction (UAE) was performed using an ultrasonic probe UP400St, with a power of 400 W and a frequency of 24 kHz (Hielscher GmbH, Teltow, Germany). In all the UAE experiments, 50% aqueous ethanol was used as the extraction solvent, and the S/L ratio was 1:20 (*w*/*v*). The experiments were performed at different amplitudes (20, 40, and 60%) at a constant extraction time of 10 min, in accordance with our previous studies [29]. Extraction was followed by filtration with a vacuum pump through the filter paper grade 34 N (thickness 0.20 mm). The obtained liquid extracts were stored at 4 °C prior to analysis. The total EY of the obtained extracts was expressed as a percentage mg/mL. UAE was applied exclusively to material pretreated with scCO_2_. The procedure was carried out at two extraction temperatures (40 °C and 60 °C) using three ultrasonic amplitudes (20%, 40%, and 60%). At 40 °C, the amplitudes of 20%, 40%, and 60% were designated as C17, C18, and C19, respectively, while at 60 °C, the corresponding amplitudes were designated as C20, C21, and C22.

### 2.6. Extraction Yield (EY)

The extraction yield was determined for all the samples by the method of evaporation under vacuum on a rotary evaporator (Devarot, Elektromedicina, Ljubljana, Slovenia). The volume of 2 mL of the extract was evaporated, and then dried in the oven at 105 °C for 3 h. Then the mass of dry extract (DE) was reported as mg of dry SCG per mL (mg/mL). The values were measured in duplicate (*n* = 2).

### 2.7. Total Phenols Content

The Folin–Ciocalteu method was used to determine the total phenols content (TPC) [30]. The content was measured by the spectrophotometric method (6300 Spectrophotometer, Jenway Ltd., Felsted, Essex, UK) at the wavelength of 750 nm. The measured content was expressed as g gallic acid equivalent (GAE)/g of the initial material SCG. All the experiments were performed in triplicate (*n* = 3).

### 2.8. ABTS Assay

The ABTS · + scavenging assay is a colorimetric assay that measures the relative ability of antioxidants to neutralize ABTS · + generated in an aqueous phase, compared to a Trolox (water-soluble vitamin E analog) standard. ABTS · + is formed when the ABTS salt reacts with a strong oxidizing agent (e.g., potassium persulfate or K_2_S_2_O_8_) and a bright green-blue solution is formed that absorbs at 734 nm. In the presence of antioxidant compounds, the solution of ABTS · + is decolored and the absorbance is inversely proportional to the concentration of antioxidants [31]. Preparation of the buffer and aqueous solutions is described below.

(1) Phosphate buffer/PBS (pH = 7.4): 8 g NaCl, 2 g KCl, 11.5 g Na_2_ HPO_4_, 2 g KH_2_PO_4_ in 1000 mL of water
(2) ABTS aqueous solution (7 mM): 0.0192 g of ABTS powder in 5 mL of water (solution A)
(3) K_2_S_2_O_8_ aqueous solution (140 mM): 0.1892 g of K_2_S_2_O_8_ powder in 5 mL of water (solution B)
(4) To prepare the solution of the ABTS+ free radical, mix 5 mL of solution A with 0.088 mL (88 μL) of solution B based on the protocol of Re et al. [32].

After 16 h (overnight), this solution is appropriately diluted with phosphate buffer (pH = 7.4) so that the absorbance (UV-Vis spectrophotometer) of the new solution is 0.700 ± 0.02 at 734 nm. To measure the absorbance at 734 nm, 4 mL of the radical solution (time 0) is transferred to a cuvette. Then 40 µL of the extract is added (diluted if necessary), the mixture is mixed, and after 6 min, the absorbance is measured again. The antioxidant capacity was determined by the equation (Equation (1)) of the calibration curve of the standard compound Trolox as mM Trolox Equivalents (mM TRE). The final results were measured in duplicate (*n* = 2) and expressed as μmol TRE/g dry plant material.

y = 39.007x + 4.4696, R^2^ = 0.9985 (1)

where y = % inhibition.

The percentage inhibition was calculated using the following equation:

% Inhibition = [(Abs_734_ (t = 0 min) − Abs_734_ (t = 6 min)) × 100]/Abs_734_ (t = 0 min)

and x = nmol TRE. The final results are expressed as μmol TRE/g of dry plant material.

### 2.9. Analysis of the Fatty Acids Methyl Esters (FAMEs)

The fatty acid profile was determined through the use of a gas chromatography system (TRACE GC 2000 Series, Thermo Quest CE Instruments, Rodano, Milan, Italy.) coupled with a flame ionization detector (FID) equipped with an autosampler (TRISPLUS AS Thermo Quest CE Instruments), after transesterification for the fatty acid methyl esters (FAMEs) preparation, as described by Vasilopoulos et al. (2022) [33]. FAMEs were analyzed on a BPX70 GC column (30 m length, 0.32 mm i.d., 0.25 μm film thickness, SGE Analytical Science, Melbourne, Australia). Helium was used as the carrier gas at a flow rate of 2.0 mL/min. The injector port and the detector temperature were maintained at 250 °C. The split ratio was 1:20. The column oven was initially set at 46 °C for 2 min, then increased to 130 °C at a rate of 50 °C/min for 10 min, then increased to 175 °C at 2 °C/min and maintained at that temperature for 2 min, then increased to 200 °C at 3 °C/min and maintained at the temperature for 3.5 min, before increasing to a plateau of 240 °C at a rate of 10 °C/min for 5 min. The total run time was 60 min. The chromatograph peaks were identified using an external 37-component standard (AccuStandard, New Haven, CT, USA) by comparing the retention time of the standards with those of the samples under the same operating time. Chromatograms were acquired and processed with the aid of Chrom Quest 5.0 software (ver. 3.2.1. Thermo Separation Products, San Jose, CA, USA). All the experiments were performed in triplicate (*n* = 3).

### 2.10. Determination of Caffeine Content by RP-HPLC-DAD

Caffeine content was quantified using reverse-phase high-performance liquid chromatography coupled with a diode array detector (RP-HPLC-DAD), as described by Kyriakoudi et al. [34]. The analytical system comprised an Agilent 1260 Infinity II Quaternary Pump VL, an Agilent 1260 Infinity II Autosampler, and a High Sensitivity Diode Array Detector (DAD) from the same series (Agilent Technologies, Santa Clara, CA, USA). Chromatographic separation was performed on an InfinityLab Poroshell 120 EC-C18 column (4 μm, 150 × 4.6 mm i.d.; Agilent Technologies). The column temperature was maintained at 30 °C. The mobile phase consisted of water with 0.1% acetic acid (solvent A) and methanol with 0.1% acetic acid (solvent B). The gradient elution program was as follows: 0 min, 30% B; 0–5 min, linear increase to 45% B; 5–15 min, linear increase to 65% B; 15–18 min, increase to 100% B; 18–25 min, return to 30% B; 25–30 min, equilibration at 30% B. The flow rate was set at 0.5 mL/min, and the injection volume was 10 μL. Sample extracts were appropriately diluted and filtered through 0.45 μm PTFE syringe filters (Frisenette, Knebel, Denmark) prior to analysis. UV detection was performed over a wavelength range of 190–600 nm. Chromatographic data acquisition and processing were carried out using OpenLab CDS software, version 3.5 (Agilent Technologies, 2021). Caffeine peaks were identified by comparing retention times and UV spectra (absorption maxima) with those of standards. Quantification was performed at 276 nm by integrating peak areas and using an external calibration curve generated from serial dilutions of caffeine standards in methanol. The calibration equation was y = 6.2165x − 151.89, with a correlation coefficient (R^2^) of 0.9993, covering the range of 200–1600 ng per 10 μL injection. The caffeine concentration is calculated as mg of caffeine per g of dry SCG (mg/g), while the measurements were performed in duplicate (*n* = 2).

## 3. Results and Discussion

### 3.1. ScCO_2_ Extraction

The recovery of non-polar and low-polar constituents from SCG was performed by using scCO_2_ extraction. In order to evaluate the extraction conditions, the impact of two different temperatures (40 °C, 60 °C) on the EY was observed, while the other parameters, pressure (250 bar) and CO_2_ flow, were kept constant (0.3 kg/h).

Based on Table 1, it is evident that a higher EY (8.50%) was achieved during the isolation of bioactive compounds from SCG at a lower process temperature (40 °C). Conversely, the application of 60 °C resulted in a lower EY, specifically 7.97%. Given that the extraction pressure (250 bar) and extraction time (4 h) were held constant throughout the process, it can be concluded that varying the temperature during scCO_2_ extraction yields different EY values. Considering the relatively small difference between the obtained yields, the results indicate that temperature had only a minor effect on the EY of SCG under the investigated conditions. On the other hand, the effect of temperature, followed by pressure changes, on the EY of SCG oil using scCO_2_ was also noted by Coelho et al. [35]. They demonstrated that at 200 bar and 300 bar, the EY decreases with increasing temperature, whereas the effect of temperature on the EY at 400 bar is not observed. They explain that this trend may result from a balance between two opposing effects: an increase in temperature reduces the density of scCO_2_, and consequently its solubility capacity, but simultaneously increases the vapor pressure of the compounds, which subsequently enhances their solubility in the supercritical fluid.

By comparing the results achieved at 40 °C in the total time of extraction of 4 h for sample C1 (8.50%) and the fractionation results (C3–C6), the table shows similar EY in total (8.75%), with the highest EY achieved in the 2nd hour of extraction (2.44%) and the lowest EY (1.95%) in the 4th hour of extraction. On the other hand, the total EY for 60 °C and the full duration of extraction (4 h) was 7.97%, while the fractionation EY (C8–C10) was 9.08%, which is higher than the total EY at 60 °C, and this can be caused by differences in the sample or the traces of material left in the scCO_2_ system. As previously mentioned, the impact of temperature on the extraction efficiency had only a minor effect on the EY of SCG under the investigated conditions. However, as a valuable conclusion from the fractionation experiments, it should be noted that depending on the target of the extraction procedure, with higher EY in the 2nd hour of extraction at both 40 °C and 60 °C, the extraction can be performed even in a reduced period of time, and at the same time the ecological and economic goals could be achieved.

### 3.2. Fatty Acids Methyl Esters Profile from SCG Extracts

**Figure 1:**
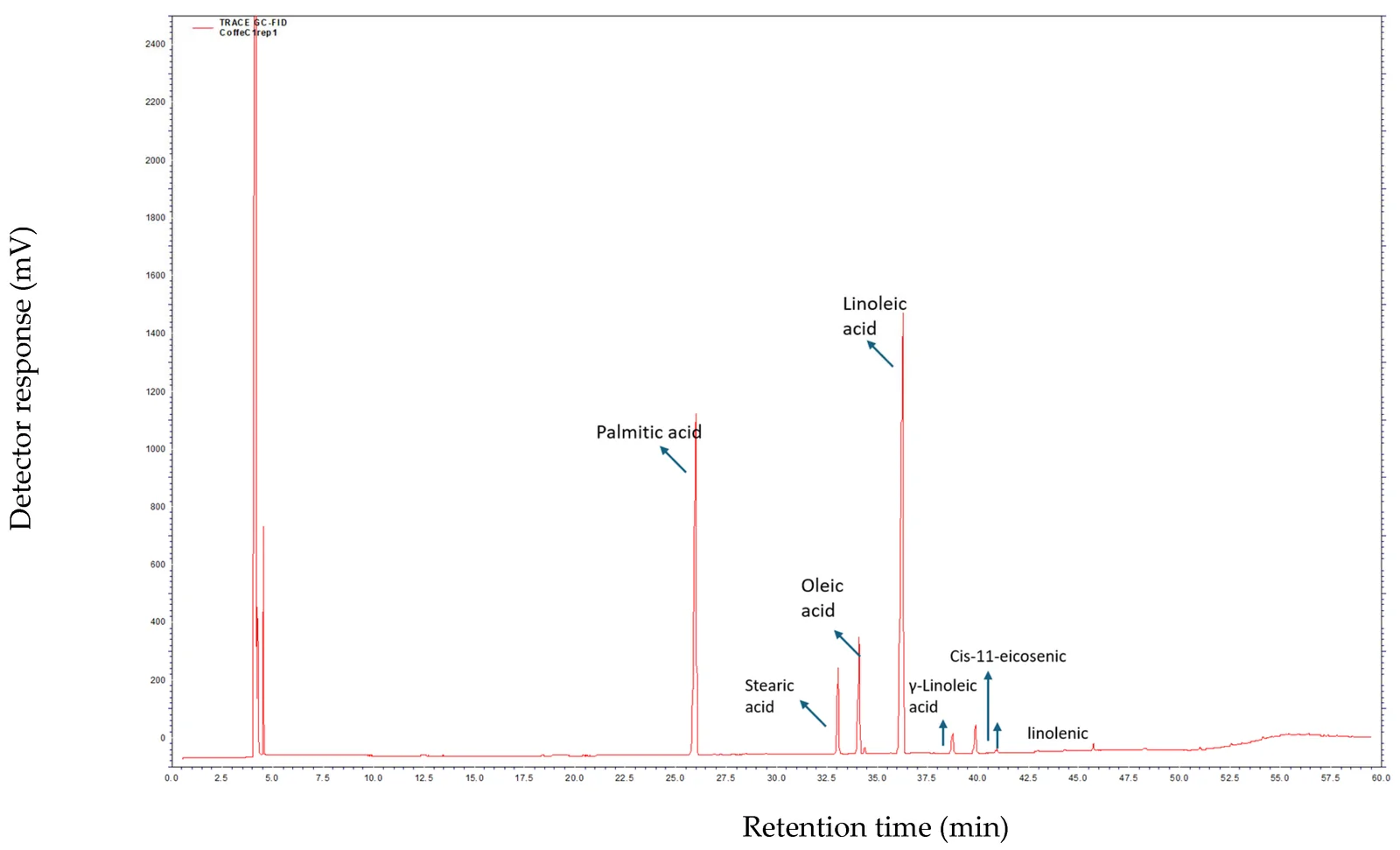
GC-MS chromatogram of the sample C1 (scCO2, 250 bar, 40 °C).

SCG contains large amounts of organic compounds, such as fatty acids, lignin, cellulose, hemicellulose, and other polysaccharides [9], that can be exploited as a source of value-added products. In our study, the lipid yield obtained by Soxhlet was 12.6% on average over two replicates, while a lower yield was recovered by scCO_2_ at 40 °C (8.50%) and scCO_2_ at 60 °C (7.97%). The Soxhlet extraction lasted 2 h longer than scCO_2_ and used hexane as the solvent instead of eco-friendly carbon dioxide. Lipid content in SCG was reported to be approximately 2% by Ballesteros et al. [36], whereas other studies have documented values as high as 13–17% [37,38]. This discrepancy could be due to different applied extraction methods and species of coffee. The predominance of linoleic acid (43.79–54.15%) and palmitic acid (32.73–45.85%) observed in all the scCO_2_ SCG oils (Figure 1) is in agreement with previous studies, which consistently identified these fatty acids as the major constituents of SCG lipids [39,40,41]. The oleic acid C18:1 (8.2–9.25%) and stearic acid C18:0 (5.84–7.81%) were reported in significantly lower shares, while the results also yielded evidence of eicosenoid and ɣ-linolenic acids that were present at below 3% of the oil and below 1%, respectively. In the present study, polyunsaturated fatty acids were found in higher concentrations (46.74–54.15%), followed by saturated fatty acids (40.45–45.84%) and monounsaturated fatty acids (8.22–12.00%). The Soxhlet extracts also showed palmitic acid (35.67 ± 2.92%) as the most dominant. The composition of SCG fatty acids in supercritical (C1-C10) and Soxhlet (C11) extracts is presented in Table 2. As can be concluded from Table 2, different extraction techniques did not alter the fatty acid profile.

### 3.3. Extraction Yield, TPC and ABTS from SCG Extracts

Once the scCO_2_ extraction was done, the material left over was further processed by UAE and SWE. Those extracts were analyzed in terms of EY, TPC, and antioxidant activity. The EY were determined in five SWE (C12, C13, C14, C15 and C16), six ultrasonic (C17, C18, C19, C20, C21 and C22) SCG extracts (Table 3) and Soxhlet (C11) SCG extracts. The highest EY were found in subcritical SCG extracts obtained at 200 °C (C16) (15.38 mg/mL) and 180 °C (13.20 mg/mL) (C15), while the lowest was in subcritical extracts obtained at 120 °C (C12) and 140 °C (C13). This can be attributed to the significant changes in the physicochemical properties of water with increasing temperature. Under subcritical conditions, the dielectric constant and viscosity of water decrease, while diffusivity increases, enhancing solvent penetration into the biomass matrix and improving mass transfer. Higher temperatures also promote the solubilization of less polar compounds and facilitate the hydrolysis of cell wall polysaccharides, resulting in the release of additional extractable material [25,26,42,43]. TPC ranged between 21.29 and 54.09 mg GAE/g with maximal values achieved in C15 (54.09 mg GAE/g) and C16 (39.94 mg GAE/g) obtained at higher temperatures of 180 and 200 °C, respectively. Previous studies indicated that the TPC were between 20 and 30 mg GAE/g SCG [44], while the antioxidant capacity of SCG ranged from 74.57 to 172 μmol Trolox equivalents/g SCG [45]. In our study, C15 proved to have a higher TPC value of 54.09 mg GAE/g and, consequently, a higher ABTS of 260.65 μmol Trolox equivalents/g SCG, which indicates a stronger overall radical-scavenging activity than in past studies. The UAE provided almost the same EY (around 3%) for all six extracts regarding applied temperatures and amplitudes. The EY, TPC and ABTS values of SCG extracts obtained by SWE (C12–C16) and UAE (C17–C22) are presented in Table 3.

### 3.4. Caffeine Content from SCG Extracts

One of the most valuable properties of SCG is its high content in caffeine, a purine alkaloid, and phenolic compounds, such as chlorogenic acid. Caffeine is widely recognized as the most frequently consumed pharmacologically active substance worldwide, primarily due to the extensive consumption of coffee and other caffeinated beverages. In addition, it is commonly used as an adjuvant analgesic in combination with drugs such as acetaminophen, aspirin, and ibuprofen [46].

**Figure 2:**
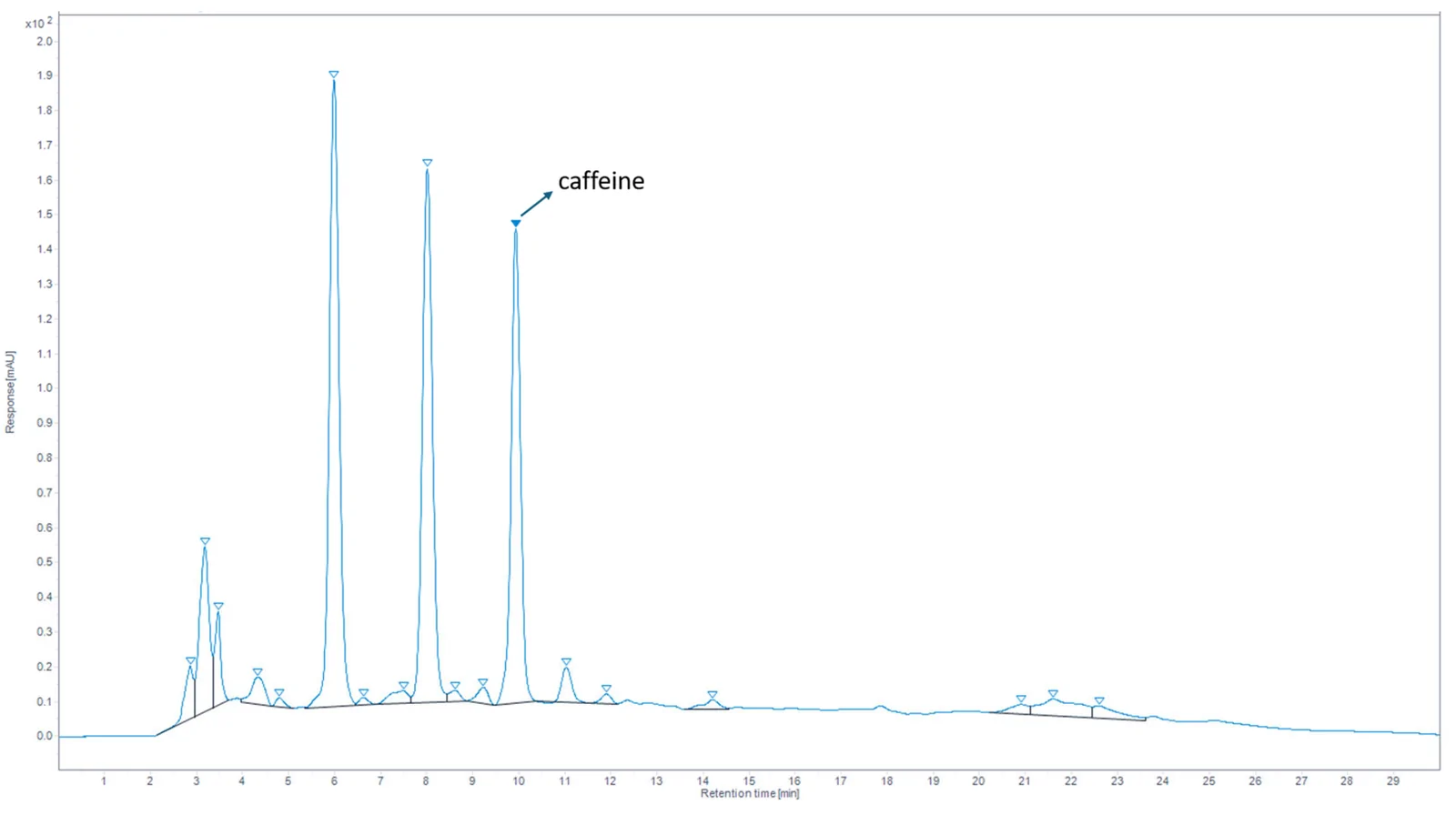
HPLC-DAD chromatogram for the sample C15, obtained by SWE at 180 °C.

Caffeine concentration in roasted coffee typically ranges from 0.8 to 1.4 g/100 g in Coffea Arabica and 1.8–2.5 g/100 g in Coffea Robusta. However, the levels in SCG are substantially lower due to its extraction taking place during beverage preparation. Nevertheless, the residual caffeine content in SCG can vary widely, with reported values ranging from 0.2 to 0.8 g/100 g depending on extraction conditions, processing and origin [47]. In our study, the highest caffeine concentration (10 mg/g) was detected in C15 (Figure 2) SWE extract obtained at 180 °C after scCO_2_ treatment. In accordance with the reported high efficiency in recovery of polyphenolic compounds (Table 3), the 180 °C extraction temperature contributed to the recovery of the polar content of defatted SCG. C15 contributed to higher polyphenolic and antioxidant content (Table 3). Shang et al. [48] observed a caffeine extraction efficiency of around 7.45–3.22 mg/g from different varieties of Arabica spent coffee grounds by the use of pressurized liquids. The range in efficiency supported the effect of the variety on the final yield. ScCO_2_ is a classic technique to remove caffeine from coffee and other materials. However, when the base material is dry, scCO_2_ has limited solubility for polar compounds. Therefore, the combination with more efficient green techniques can contribute to the total valorization [24,49]. In this way, in the first step, the non-polar compounds were isolated, and then the extract rich in caffeine were obtained. Shalmashi et al. [50] also explored how caffeine was extracted from tea waste using subcritical water as a green solvent. It examines the effects of temperature, flow rate, particle size, and pressure on the EY, finding that at 175 °C and the evaluated conditions, caffeine was successfully recovered from tea waste. Similarly, to our study, several studies have demonstrated that subcritical water is an efficient green solvent for caffeine extraction from coffee matrices, with the isolated concentration strongly dependent on temperature and residence time [51]. The possible explanation is that high-temperature water can disrupt hydrogen bonding and weaken interactions between caffeine and the cellular matrix, promoting desorption and diffusion into the extraction medium. In some cases, partial matrix swelling or cell wall disruption further enhances caffeine extraction. The caffeine concentration in all the UAE extracts was around 4 mg/g no matter the different temperatures and amplitudes applied. The results of caffeine contents obtained under various extraction conditions through SWE and UAE are summarized in Table 4. Performing UAE of light–medium roast SCG at 60% amplitude, a similar concentration of caffeine (400.1 mg/100 g) was achieved in the study of Silva et al. [52]. The obtained values confirm the advantage of UAE in SCG caffeine isolation in comparison to conventional extraction approaches [53].

## 4. Conclusions

In the present study, the total utilization of the initial material, SCG, was evaluated. The first step of the process involved scCO_2_ isolation of nonpolar and low-polar compounds, including lipids and fatty acids. The next step included isolation of polar compounds by applying UAE and SWE. All of the applied techniques showed benefits depending on the target compounds, confirming the advantage of the integration of various extraction techniques in terms of utilizing the food waste material. The highly favorable FAME profile, characterized by a predominance of polyunsaturated fatty acids, particularly linoleic acid, suggests that the valorization of SCG collected from local cafés and restaurants proved to be a promising approach for the sustainable recovery of high-value bioactive compounds and lipids with potential applications in the cosmetic, oleochemical, and biofuel industries.”

## Figures and Tables

**Table 1:** Extraction yields achieved during fractionation at 40 °C and 60 °C.

40 °C	EY (g)	EY (%)	60 °C	EY (g)	EY (%)
4 h extraction at 40 °C			4 h extraction at 60 °C		
C1	4.25	8.50	C2	3.98	7.97
Fractionation at 40 °C			Fractionation at 60 °C		
C3	1.11	2.22	C7	1.15	2.30
C4	1.22	2.44	C8	1.15	2.30
C5	1.07	2.14	C9	1.13	2.26
C6	0.98	1.95	C10	1.08	2.16
* Ƹ EY	4.38	8.75	* Ƹ EY	4.51	9.02

* Ƹ represents the sum of extraction yield achieved during fractionation.

**Table 2:** Fatty acid composition of SCG in scCO_2_ (C1–C10) and Soxhlet (C11) extracts.

	C1	C2	C3	C4	C5	C6	C7	C8	C9	C10	C11
**Palmitic (C16:0)**	35.69 ± 0.49	45.85 ± 0.49	37.53 ± 0.05	34.95 ± 0.17	37.40 ± 0.47	31.98 ± 0.07	38.21 ± 0.16	36.24 ± 0.31	34.64 ± 0.54	34.08 ± 0.16	35.67 ± 2.92
**Stearic (C18:0)**	6.52 ± 0.03	* ND	5.87 ± 0.07	6.05 ± 0.02	6.20 ±0.09	7.81 ± 0.01	5.84 ± 0.11	6.49 ± 0.04	6.45 ± 0.05	7.09 ± 0.01	7.71 ± 0.04
**Oleic (C18:1 cis ω9)**	8.63 ± 0.08	* ND	8.22 ± 0.08	8.46 ± 0.02	8.21 ± 0.08	9.25 ± 0.03	8.48 ± 0.09	8.55 ± 0.04	8.58 ± 0.07	8.84 ± 0.03	8.99 ± 0.01
**Linoleic (C18:2 cis ω6)**	45.47 ± 0.32	54.15 ± 0.49	48.38 ± 0.09	47.64 ± 0.12	48.18 ± 0.30	46.95 ± 0.06	47.47 ± 0.04	45.39 ± 0.17	46.23 ± 0.36	46.14 ± 0.09	14.42 ± 0.89
**ɣ-Linolenic (C18:3 cis ω6)**	1.50 ± 0.02	* ND	* ND	1.26 ± 0.04	* ND	1.38 ± 0.01	* ND	1.42 ± 0.03	1.51 ± 0.02	1.50 ± 0.02	1.40 ± 0.05
**cis-11-Eicosenoic (C20:1 cis ω9)**	1.99 ± 0.04	* ND	* ND	1.64 ± 0.03	* ND	2.51 ± 0.04	* ND	1.90 ± 0.05	1.92 ± 0.03	2.14 ± 0.04	3.01 ± 0.02
**Linolenic (C18:3 trans ω3)**	0.19 ± 0.02	* ND	* ND	* ND	* ND	0.18 ± 0.00	* ND	* ND	0.18 ± 0.01	0.21 ± 0.01	0.26 ± 0.01
**Saturated**	42.22 ± 0.47	45.85 ± 0.49	43.40 ± 0.11	40.99 ± 0.19	43.61 ± 0.38	39.79 ± 0.07	44.05 ± 0.05	42.74 ± 0.27	41.09 ± 0.53	41.17 ± 0.15	40.87 ± 0.59
**Monounsaturated**	10.62 ± 0.12	-	8.22 ± 0.08	10.10 ± 0.05	8.22 ± 0.08	11.76± 0.04	8.48 ± 0.09	10.45 ± 0.08	10.50 ± 0.08	10.98 ± 0.04	11.99 ± 0.01
**Polyunsaturated**	47.16 ± 0.35	54.15 ± 0.49	48.38 ± 0.09	48.91 ± 0.14	48.18 ± 0.30	48.45 ± 0.04	47.47 ± 0.04	46.81 ± 0.18	48.41 ± 0.58	47.84 ± 0.11	17.15 ± 0.57
**Total ω3**	0.19 ± 0.02	* ND	* ND	* ND	* ND	0.18 ± 0.00	* ND	* ND	0.18 ± 0.01	0.21 ± 0.01	0.26 ± 0.01
**Total ω6**	46.97 ± 0.34	54.15 ± 0.49	48.39 ± 0.09	48.91 ± 0.14	48.18 ± 0.30	48.39 ± 0.08	47.47 ± 0.04	46.81 ± 0.19	47.80 ± 0.30	47.64 ± 0.11	15.82 ± 0.94

* ND refers to not detected.

**Table 3:** EY, TPC and ABTS.

Sample	EY (mg/mL)	TPC (mg GAE/g)	ABTS (μmol TE/g)
C12	1.55	21.76 ± 0.57	163.75 ± 7.90
C13	1.50	21.29 ± 0.83	119.59 ± 5.16
C14	4.80	34.62 ± 1.10	250 ± 1.78
C15	13.20	54.09 ± 2.0	260.65 ± 20.07
C16	15.38	39.94 ± 1.09	226.65 ± 18.46
C17	3.15	26.65 ± 0.70	186 ± 5.20
C18	3.28	25.12 ± 0.68	110.52 ± 9.61
C19	3.08	24.74 ± 1.15	140.57 ± 7.25
C20	3.65	26.96 ± 1.18	142.69 ± 11.31
C21	3.62	25.67 ± 1.08	175.02 ± 10.09
C22	3.55	27.14 ± 0.97	147.72 ± 6.23

**Table 4:** Caffeine content in SCG extracts obtained by SWE (C12–C16) and UAE (C17–C22).

Sample	Concentration (mg/g)
C12	1.87 ± 0.02
C13	1.37 ± 0.03
C14	2.37 ± 0.04
C15	10.01 ± 0.51
C16	4.09 ± 0.03
C17	4.82 ± 0.11
C18	4.01 ± 0.14
C19	4.21 ± 0.02
C20	4.85 ± 0.12
C21	4.98 ± 0.03
C22	4.15 ± 0.22

## Data Availability

The original contributions presented in the study are included in the article; further inquiries can be directed to the corresponding author.
